# Preparation and Characteristics of Ball-Milled Blueberry Peel Particles and Their Application in Ice Cream

**DOI:** 10.3390/foods13223660

**Published:** 2024-11-17

**Authors:** Li-Hua Pan, Jia-Hui Lin, Mei-Jia Li, Lei Cao, Xiao-Yu Liu, Yuan-Yuan Deng, Shui-Zhong Luo, Zhi Zheng

**Affiliations:** 1 School of Food and Biological Engineering, Hefei University of Technology, Hefei 230009, China; panlihua@hfut.edu.cn (L.-H.P.); katyalin@126.com (J.-H.L.); limeijia0805@163.com (M.-J.L.); chloeliu0506@163.com (X.-Y.L.); zhengzhi@hfut.edu.cn (Z.Z.); 2 Institute of Agro-Products Processing, Anhui Academy of Agricultural Sciences, Hefei 230031, China; leicao1008@163.com; 3 Sericultural & Agri-Food Research Institute Guangdong Academy of Agricultural Sciences/Key Laboratory of Functional Foods, Ministry of Agriculture and Rural Affairs/Guangdong Key Laboratory of Agricultural Products Processing, Guangzhou 510640, China; yuanyuan_deng@yeah.net; 4 Anhui Province Key Laboratory of Agricultural Products Modern Processing, Hefei University of Technology, Hefei 230009, China

**Keywords:** blueberry peels, ball milling, ice cream, Pickering emulsion

## Abstract

Ice cream is popular but contains high amounts of saturated fats and few health-promoting ingredients. In the presence of xanthan gum (0.25%), blueberry peel particles prepared through ball-milling treatment (BMPs) were used to prepare ice cream containing camellia oil as a fat replacer. The BMPs possessed smaller particle sizes, larger contact angles, and higher contents of anthocyanin aglycone compared with commonly milled blueberry peel particles. BMPs with the largest contact angle (66.30°) were obtained by ball-milling the blueberry peel at 15 Hz for 6 h (BMP_15Hz6h_). The ice cream mixes were depicted as linear viscoelastic gel-like solids, and their apparent viscosity, G′ and G′, increased with the increase in the BMP_15Hz6h_ concentration. Ice cream with strong antioxidant activity and good freeze–thaw stability was acceptable and desirable in the presence of 0.5% BMP_15Hz6h_.

## 1. Introduction

Ice cream is a popular dessert that is made of milk products, fat, sweeteners, emulsifiers, stabilizers, and condiments, and it undergoes many processing steps, including the mixing, sterilizing, and homogenizing of all the components (namely, preparing the ice cream mix) and subsequent aging, freezing, and hardening [1]. An ice cream mix exhibits an oil-in-water emulsion, where proteins, fat globules, ice crystals, air bubbles, and particle stabilizers are dispersed in the continuous phase containing milk proteins, sugars, salts, emulsifiers, and hydrocolloids, and is responsible for the stability and texture of the ice cream [1]. However, an ice cream mix contains a high amount of saturated fatty acids, so the high consumption of ice cream will increase the risk of obesity, cardiovascular disease, and other health problems [2]. Therefore, the industry and consumers are continuously seeking alternative ingredients to meet the demand for new and healthier ice cream.

To reduce the amount of saturated fat in ice cream, liquid oil with high contents of natural unsaturated fat, such as sunflower oil [3], rapeseed oil [4], and peanut oil [5], has been incorporated into ice cream mixes after it has been structured into an oleogel in the presence of Pickering gelators and/or hydrocolloids, which make the rheological behavior of the liquid oil similar to that of solid crystalline fat. In addition, the by-product peel powder from pitaya [6], pomegranate [7], and persimmon [8] has also been added into ice cream mixes to produce functional ice cream with dietary fibers, polyphenols, and/or antioxidants.

Blueberries, considered “super fruits”, are able to scavenge free radicals and prevent cardiovascular disease, neurological decline, and type 2 diabetes mellitus, which is predominantly attributed to their abundant phytochemicals and especially to their rich anthocyanin pigments [9]. However, blueberry anthocyanins are mainly located in the peel, which contains up to 90% of the anthocyanins of the whole fruit, and they are not easily extracted, owing to their binding to cell walls [10]. In addition, blueberry peel, which accounts for nearly 30% of the dry weight of the whole blueberry, is usually discarded due to its low solubility and immiscibility. Therefore, it is of importance to find an efficient method to utilize blueberry peel for the development of blueberry planting and processing enterprises.

Ball-milling technology has been used for the production of ultrafine powders and foods, wherein medium balls colliding in the ball mill tank result in materials being modified through the creation of shear force and stress [11,12,13]. Particles on the nanometer/submicron scale from fruit peel, such as yam [14] and onion peel [15], have been obtained using a ball mill grinder. Excitingly, whole red rice materials were media-milled into nano/submicrometer particles that can used as stabilizers for Pickering emulsions with intrinsic antioxidant properties [16]. Therefore, modification of the immiscibility of blueberry peel during ball milling could realize its full utilization and enlarge its application scope.

Camellia oil from tea seeds (*Camellia oleifera* Abel.) is a unique edible vegetable oil with high contents of oleic acid and tea polyphenols and can reduce blood lipid levels and prevent coronary heart disease [17]. In our previous work, camellia oil-based oleogels were structured in the presence of xanthan gum [17] and used as fat replacers to prepare functional cookies [18,19] and soft candies [20], which reduced the contents of saturated fatty acids and trans fatty acids in the cookies and increased the content of polyunsaturated fatty acids. The aims of this study are to (1) prepare blueberry peel particles using ball-milling treatment at a low temperature, (2) evaluate the physicochemical characteristics of the ball-milled blueberry peel particles, and (3) investigate the possibility of the preparation of ice cream using camellia oil instead of solid fats and using blueberry peel particles as stabilizers in the presence of xanthan gum.

## 2. Materials and Methods

### 2.1. Materials and Chemicals

Fresh blueberry (Britewell) fruits were provided by Anhui Huiwang Food Co., Ltd. (Hefei, China). Nile red, 3′,6′-dihydroxy-5-isothiocyanato-3H-spiro[isobenzofuran-1,9′-xanthen]-3-one (FITC), 6-hydroxy-2,5,7,8-tetramethylchroman-2-carboxylic acid (Trolox), gallic acid (GA), and cyanidin-3-O-glucoside (CGE) were obtained from Solarbio Science & Technology Co., Ltd. (Beijing, China). All other chemicals used were analytical-grade and bought from Sinopharm Chemical Reagent Co., Ltd. (Shanghai, China).

### 2.2. Preparation of Blueberry Peel Particles

The fresh blueberry fruits were frozen at −38 °C for 6 h, placed in tap water for about 10 s, and peeled by hand immediately. The peel was collected and then freeze-dried at −38 °C for 24 h in an FD-1 B-50 freeze-dryer (China Beijing Boyikang Experimental Instrument Co., Ltd., Beijing, China) [10]. The dried blueberry peel was ground with a QSB-200 crusher (Yongkang Industry & Trade Co., Ltd., Yongkang, China) for 3 min and passed through a 60-mesh sieve to yield the commonly milled blueberry peel particles (CMPs). CMPs (4 g) were mixed with ZrO_2_ spheres (2 cm in diameter) at a mass ratio of 1:4 and put into the sample chamber, which was intermittently laid in a liquid nitrogen tank filled with liquid nitrogen during the ball-milling treatment to maintain the temperature at 4–8 °C. They were then placed into a BM500 cryogenic mill (Anton-paar, Shanghai, China), and ball-milled at 15 Hz or 25 Hz for 1.5 h, 3 h, and 6 h, respectively, to yield the ball-milled blueberry peel particles (BMPs) [15], denoted as BMP_15Hz1.5h_, BMP_15Hz3h_, BMP_15Hz6h_, BMP_25Hz1.5h_, BMP_25Hz3h_, and BMP_25Hz6h_. The blueberry peel CMPs and BMPs were separately stored at −18 °C in double Ziploc bags for further use.

### 2.3. Property Analysis of Blueberry Peel Particles

#### 2.3.1. Size Distribution

The particle size distribution was determined at room temperature using the wet technique [21]. The deionized water was added into the sample tank, which connects with a BT-601 circulating disperser. The blueberry peel particles were continuously added into the sample tank and dispersed via ultrasonic treatment for 3 min. When the shading rate reached 10–15%, the values of D_10_, D_50_, D_90_, and specific surface area (SSA) of samples were recorded with an MS 200 laser particle size analyzer (Malvern Instruments Limited, Worcestershire, Britain) and calculated by the software using the Rayleigh theory, representing 10%, 50%, and 90% of total volume at particle distribution, supposing a spherical shape. Meanwhile, span and Φ (cell wall breakage ratio) were calculated as follows. Span = (D_90_ − D_10_)/D_50_, Φ = 1 − (1 − 10/D_50_)^3^ (if D_50_ ≤ 10 μm, then Φ = 1).

#### 2.3.2. Wettability

The wettability was assessed by monitoring the contact angle with an SCA20 contact angle measuring instrument (DataPhysics Instruments GmbH, Stuttgart, Germany) following our previous work [17]. The blueberry peel powder (200 mg) was pressed into a thin slice with a diameter of 6.5 mm and a thickness of 2 mm and put on the sample platform. Then, 2 μL of water or camellia oil extruded through a high-precision sampler was added to the thin slice of sample. The contact angle (θ_o/w_) was determined according to the captured images of the contact sheet surface.

#### 2.3.3. Micromorphology

The microstructure was observed with a high-resolution field emission scanning electron microscope, Regulus 8230 (Hitachi Co., Ltd., Tokyo, Japan). Blueberry peel particles were carefully placed onto the conductive adhesive carbon tape affixed to the sample plate, and then the micromorphology was examined after gold was sprayed on the sample.

#### 2.3.4. Anthocyanin Composition

The anthocyanin composition of blueberry peel particles was analyzed with a UPLCOrbitrap-MS system (Thermo Fisher Scientific Instruments Co., Ltd., Shanghai, China) connected with a Waters Accuracy UPLC HSS T3 column (2.1 mm × 100 mm; Thermo Fisher Scientific Instruments Co., Ltd., Shanghai, China), according to our previous work [10].

### 2.4. Preparation of Ice Cream

The ice cream was prepared according to the method proposed by Samakradhamrongthai et al. [22], with slight modifications. The ingredients used in the ice cream formula (IC-B) consisted of skim milk, sucrose, skim milk powder, camellia oil, xanthan gum, and BMP_15Hz6h_ (Table 1). Skim milk and camellia oil were placed into a pan and heated to 50 °C in a water bath. Sucrose, skimmed milk powder, xanthan gum, and BMP_15Hz6h_ were completely premixed and then added and continuously stirred for 10 min to form a homogeneous ice cream mix. The ice cream mix was pasteurized at 85 ± 1 °C for 2 min, then homogenized with an FA25 high shear emulsifying machine (Fluko Co., Ltd., Munich, Germany) for 2 min, first at 60 °C and 10,000 rpm, sequentially homogenized at 16,000 rpm for 2 min, and then quickly chilled to 4 °C and aged at 4 °C for 24 h to ensure thorough hydration of all ingredients. The aged ice cream mixes were frozen in an ice cream maker (Hubei Dongbei Electromechanical Co., Ltd., Huangshi, China) for 6 min to 4 °C, further whipped for 7 min, and then hardened at −30 °C for 24 h to obtain ice cream samples. The ice cream samples were stored at −18 °C for further use. The ice cream with 0, 0.1%, 0.3%, 0.5%, and 1.0% BMP added was designated as IC-B_0.0%_, IC-B_0.1%_, IC-B_0.3%_, IC-B_0.5%_, and IC-B_1.0%_, respectively.

### 2.5. Rheological Behavior Analysis of Ice Cream Mix

The rheological behavior of the ice cream mix was measured with a DHR-3 rheometer (TA Instruments, Leatherhead, UK) [20]. The 2 mL ice cream mix was tiled on the plate-plate configuration (test platform) of the rheometer. The gap between the test platform and the fixture with a diameter of 40 mm was set at 1000 μm. The frequency sweep test was measured at a strain of 0.5% and a frequency range of 0.1–10 Hz. The flow measurement was performed at a shear rate of 0.001–100 s^−1^. All procedures were carried out at 25 °C.

### 2.6. Characteristic Assessment of Ice Cream

#### 2.6.1. Overrun, First Dripping Time, Melting Rate, and Firmness

The overrun, first dripping time, melting rate, and firmness of the ice cream samples were measured at 25 °C and relative humidity of 60% according to the method proposed by Liu, Wang, Liu, Wu, and Zhang [23]. A 200 mL container was weighed, and the overrun percentage was determined as follows

Overrun% = (weight of ice cream mixes − weight of frozen ice cream) ÷ weight of frozen ice cream × 100%.

The first dripping time was defined as the required time (min) for the first melted drop of the ice cream to fall. The melting rate (%/min) was calculated as the slope of the linear curve interval of the dripped portion as function of the time. The firmness was gauged with a TA.XT2 Texture analyzer (Stable Micro System, Godalming, UK), and the firmness value was obtained through Texture Expert software (version 1.20, Stable Micro Systems, London, UK). The measurement conditions for the firmness were as follows: a P/5 cylindrical probe 5 mm in diameter, 2 mm/s pre-test speed, 2 mm/s test speed, 10 mm/s post-test speed, 20 mm test distance, and 5 g trigger force [23].

#### 2.6.2. Macro- and Micro-Structure Observation

The air bubbles scattered in the ice cream were observed through a microscope with a digital camera (Nikon Co., Ltd., Tokyo, Japan) in a bright area. The distribution of the protein and fat granules in the ice cream was observed through an FV1000 confocal laser scanning microscope (Olympus Co., Ltd., Tokyo, Japan) with the help of fluorescent dyes, FITC and Nile red, respectively, at the excitation wavelengths of 633 nm and 488 nm [24].

#### 2.6.3. Physical Storage Stability

The changes in the particle size and creaming index (CI) of ice cream samples kept in a 10 mL transparent centrifuge tube with a lid and stored at 4 ± 1 °C were monitored on the 7th, 14th, 21st, and 28th day of storage. The Z-ave (Z-average size) of the ice cream droplet was gauged with a Mastersizer 2000 (Malvern Instruments Ltd., Malvern, UK), wherein the dispersant of water, particle refractive index of 1.520, particle absorption rate of 0.1, and dispersant refractive index of 1.330 were set. The CI value was calculated as follows: CI% = (Hs/He) × 100, where Hs stands for the height of the serum layer, and He indicates the height of the entire ice cream [17].

#### 2.6.4. Sensory Evaluation

Sensory properties were evaluated according to the method proposed by Liu, Wang, Liu, Wu, and Zhang [23], with some modifications. On the first and last day of 28-day storage, sensory evaluation was conducted by 30 pre-trained panelists (18 females and 12 males, ages between 20 and 55) from School of Food and Biological Engineering using a 5-point hedonic scale for five attributes (appearance, texture, taste, flavor, and overall acceptability), where 1 meant “dislike extremely” and 5 meant “like extremely” (No. HFUT20241018001, 18 October 2024, Hefei University of Technology Biomedical Ethics Committee). Overall scores were calculated as the sum of the scores of five attributes.

### 2.7. Statistical Analysis

All tests were performed in triplicate. The results were expressed as mean values ± standard error. Statistical analysis and multiple comparisons between the means were analyzed using one-way analysis of variance and LSD test at 5% level of significance using SPSS 17.0 software.

## 3. Results and Discussion

### 3.1. Effects of Ball Milling on Physical Properties of Blueberry Peel Particles

#### 3.1.1. Particle Size Distribution

The particle size has an important impact on the wettability and other characteristics of particles. The particle size of BMP was reduced by approximately 10 times due to the low-temperature ball-milling treatment compared with that of CMP, and it decreased with the increase in the ball milling time (Table 2). When the ball milling time increased from 1.5 h to 6.0 h, the D_10_ value of blueberry peel particles decreased from 30 µm to 3.5–5.5 µm, D_50_ value from 210 µm to 15.0–24.0 µm, and D_90_ value from 550 µm to 40–60 µm, respectively. Similar results of reduced particle sizes were also found by Jiang, Ramachandraiah, Wu, Li, and Eun [15] for onion peel and by Xiao, Yang, Zhao, Wang, and Ge [25] for pomelo peel. In the case of the same ball milling time, with the increase in the ball milling frequency, the particle size decreased and then increased as a whole. The larger particles measured after milling at the higher frequency may be due to the aggregation of some particles. More than 90% of blueberry peel cells were ruptured during ball milling, while only 13.6% of the cell walls were broken via common milling. As the ball milling time was extended to 6 h, the particle size of blueberry peel particles obtained at 15 Hz (BMP_15Hz6.0h_) was smaller than that obtained at 25 Hz (BMP_25Hz6.0h_). BMP_15Hz6.0h_ possessed the lowest span value and the largest Φ value, suggesting that BMP_15Hz6.0h_ had a more uniform particle size distribution and better homogeneity than other blueberry peel particles [26]. Especially, BMP_15Hz6h_ achieved 96.38% of Φ and 1.01 m^2^/g of SSA, which indicated that they serve as high-quality powdery additives due to the strong digestion possibility of nutrient components [12].

#### 3.1.2. Surface Properties

**Figure 1:**
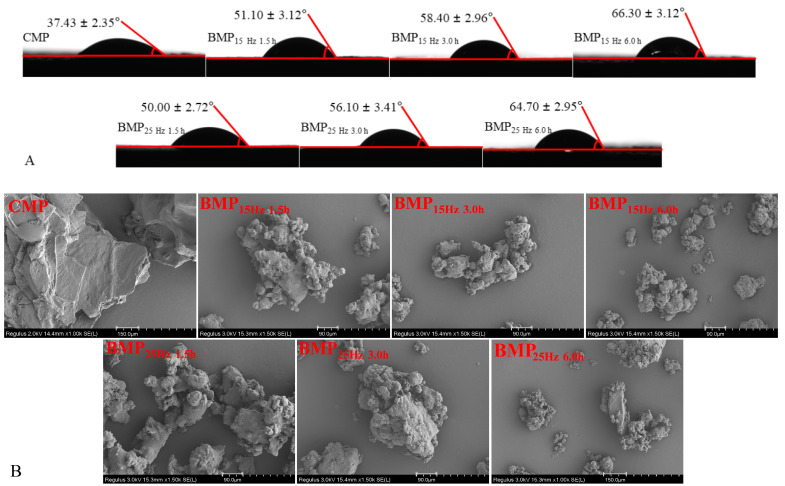
Effects of ball milling frequency and ball milling time on contact angle (**A**) and microstructure (**B**) of blueberry peel particles.

Generally, the greater the θ_o/w_ value of the test samples, the higher their surface hydrophobicity is. CMP had a θ_o/w_ of 37.43°, which is much lower than that (51.10–66.30°) of BMP (Figure 1A), which might be ascribed to the smaller particle size of BMP. Similar results were also described by Yang and Tang [27], who reported that the contact angles of okara holocellulose were obviously increased and their surface hydrophobicity was largely improved after ball-milling treatment. BMP_15Hz6h_, with an θ_o/w_ of 66.30°, are preferentially wetted by water, but they can also be wetted by oil so that they can stabilize the emulsions through a Pickering mechanism [28].

#### 3.1.3. Microstructure

CMP showed an irregular flaky structure and uneven surface, while BMP exhibited an irregular spherical form (Figure 1B), which suggested that ball-milling treatment greatly altered the microstructure of blueberry peel particles. BMP tended to gather in clusters and exhibited a highly agglomerated morphology, which may be due to the small size, large SSA, and uniform particles. Wang, Zhang, Devahastin, and Liu [12] also found that the ball-milled horseradish powder exhibited more uniform morphological properties than other milled samples. Greater modifications in the size and shape of the BMP caused by ball-milling treatment may introduce significant effects on the physicochemical and functional properties of the particles (Table 2 and Table 3).

### 3.2. Effects of Ball Milling on Anthocyanin Composition of Blueberry Peel Particles

The low-temperature ball-milling treatment slightly changed the percentage of individual anthocyanins but did not change the anthocyanin composition of blueberry peel particles (Table 3). The same 35 anthocyanins in the form of aglycone or glycoside were identified from both CMP and BMP_15Hz1.5h_, and BMP_15Hz1.5h_ had a higher percentage of anthocyanin aglycone and a lower percentage of anthocyanin glycoside than CMP, suggesting that the contents of anthocyanin aglycons in BMP were enhanced by ball milling. This may be due to the decrease in the particle size and the increase in the surface area of BMP_15Hz1.5h_, which led to the more extensive hydrolysis of the anthocyanin glycoside by the endogenous enzyme of blueberry peel, resulting in the release of anthocyanin aglycone. However, only 4 (3 aglycones and glycosides) of the 35 substances changed significantly in terms of contents after ball milling; nevertheless, these 4 anthocyanins were found at very low amounts in the peels (less than 0.03%) in terms of the proportion of total anthocyanins. The content of total anthocyanins in the dried blueberry peels was 28.05 ± 0.57 mg CGE/g DW [10]. Wang, Zhang, Devahastin, and Liu [13] reported that low-temperature ball-milling treatment accelerated the hydrolysis of substrates (i.e., glucosinolates) and increased the contents of bioactive compounds (i.e., isothiocyanates).

### 3.3. Effects of BMP on Rheological Behavior of Ice Cream Mix

**Figure 2:**
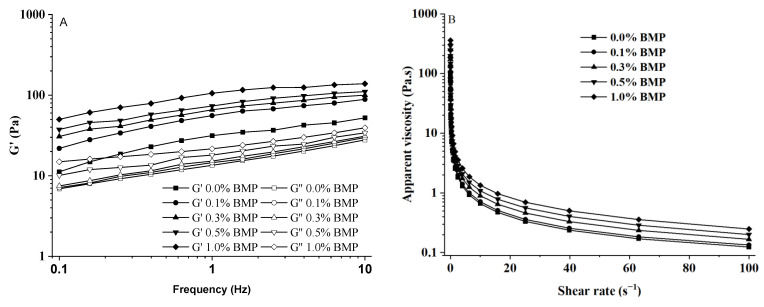
Rheological properties of ice cream mixes with different levels of BMP_15Hz1.5h_ added. (**A**), G’, storage modulus; (**B**), apparent viscosity.

With increasing levels of BMP_15Hz1.5h_, the values of storage modulus, G′, and loss modulus, G′′, of the ice cream mix increased at the same frequency (Figure 2A). The G′ value of the ice cream mix was higher than the G′′ value over the tested frequency range (Figure 2A), indicating that the ice cream mix showed mechanical elasticity. In the ice cream mix, particles, such as fats, are adsorbed on the interface, and air bubbles and small droplets interact to form gel-like emulsions [29]. Meanwhile, when BMP_15Hz1.5h_ were added into the mixing system, they intertwined with other polymers to form a gel-like three-dimensional structure, which increased the viscosity index of the mix emulsion. The amelioration of the rheological properties of the ice cream mix emulsion contributed to the improvement in the viscous structure of ice cream samples [29].

The apparent viscosity of the ice cream mix emulsions decreased with increasing shear rate (Figure 2B). The ice cream mix system showed high apparent viscosity at low shear and low viscosity at high shear owing to the breakdown of the internal network and the arrangement of ice crystals and macromolecules in the shear direction, as well as due to the specific rheological properties of xanthan gum showing pseudoplastic behavior (shear thinning) [29]. The viscosity of the ice cream mixes dramatically increased as the added levels of BMP_15Hz6h_ increased in the ice cream formula (Figure 2). This may be due to the introduction of BMP Pickering stabilizers and the increase in the fiber and hydrophilic compound contents of BMP_15Hz6h_ in the mixture, causing an increase in the apparent viscosity [30]. Reducing fat would also lead to an increase in the viscosity of ice cream, causing less creamy and harder ice cream [31]. Additionally, the abundant anthocyanins in BMP_15Hz6h_ might interact with the components of ice cream, such as proteins, unsaturated fatty acid, and xanthan gum, finally forming more stable gel networks and effectively increasing the viscosity of ice cream [32].

### 3.4. Effects of BMP on Physicochemical Properties of Ice Cream

#### 3.4.1. Overrun and Firmness

The addition of BMP_15Hz6h_ significantly changed the overrun and firmness of IC-B samples (Table 4). As the added levels of BMP_15Hz6h_ increased, the overrun value of the IC-B firstly increased, reaching the highest value of 45.13% (at 0.5% BMP_15Hz6h_ addition), then decreased to 38.22% (at 1.0% BMP_15Hz6h_ addition), which is still higher than that of the control sample IC-B_0.0%_ with an overrun value of 32.99%. The overrun values of blueberry pulp-added ice cream were reported as 36.21–39.66% [33], which is lower than that of IC-B_0.5%_. According to the results of Şentürk, Akın, Göktepe, and Denktaş [34], the addition of 1–3% blueberry puree into the probiotic ice cream caused no significant effects on overrun values of samples (*p*  > 0.05). Similar to our results, the addition of peanut oil bodies as a fat replacer into ice cream [5] had a higher overrun value. It has been considered that the overrun value is affected by the contents of fat globule, protein, and emulsifier in the ice cream mix and the viscosity in the production system [35]. The increased overrun value of BMP_15Hz6h_-added ice cream could be ascribed to the reduced fat and the Pickering emulsifier attribute of BMP, as well as the interaction of the abundant anthocyanin in BMP_15Hz6h_ with proteins.

Oppositely, the firmness of IC-B samples first decreased, obtaining the lowest value of 19.03 N (at 0.5% BMP_15Hz6h_ addition), and then increased to 24.41 N (at 1.0% BMP_15Hz6h_ addition), which is still lower than that of the control sample IC-B_0.0%_ with the highest firmness of 33.15 N. The IC-B samples in the present work had lower firmness than the camel milk ice cream with blueberry fruits (137–203 N, 33) and the probiotic ice cream with blueberry puree (5000–8000 N, 34) but showed similar hardness to the ice cream with persimmon peel [8]. The firmness of ice cream is a function of the composition in ice cream formulation (protein, fat, sweetener, hydrocolloids, etc.), ice crystal sizes, ice crystal phase volume, system viscosity, the size and distribution of air bubbles, and the fat instability [36]. The compounds that create the network improve the air state (Figure 2) and decrease the ice crystal size [37], therefore reducing the firmness of IC-B samples. Additionally, the firmness of ice creams may be ascribed to the overrun. It has been reported that the higher the overrun value of ice cream, the less hard it is, because the presence of more air in the network of ice cream makes it easy for the probe of the texture analyzer to penetrate [8].

#### 3.4.2. Melting Behavior

The addition of BMP_15Hz6h_ significantly improved the melting resistance of ice cream (Table 4). As the added levels of BMP_15Hz6h_ increased, the first dripping time quickly prolonged, reaching the maximum value of 20.18 min (at 0.5% BMP_15Hz6h_ addition), then slightly shortened to 18.00 min (at 1.0% BMP_15Hz6h_ addition), which is still longer than the 14.16 min of the IC-B_0.0%_ sample without BMP_15Hz6h_. Also, the melting rate of the BMP-added IC-B samples decreased from 3.13%/min to 2.71%/min.

Too quick of melting causes it to lose its edible attribute and become susceptible to thermal shock, while too slow of melting is considered a trait of an inferior ice cream product. The meltdown rate reflects both fat contents and rheological properties of ice cream; the higher the contents of fats and the greater the viscosity, the lower the melting rate [35,38]. In the present work, the high viscosity created by xanthan gum and BMP_15Hz6h_ is responsible for the reduced meltdown rate and the prolonged first dripping time. Similar to our results, the addition of pomegranate peel [7] and persimmon peel [8] resulted in a significant increase in the melting resistance of ice cream. The first dripping time of camel milk ice cream containing blueberry fruits (26–29 min) was also longer than that of the control without blueberry fruits (23.1 min) [33].

#### 3.4.3. Antioxidant Activities

**Figure 3:**
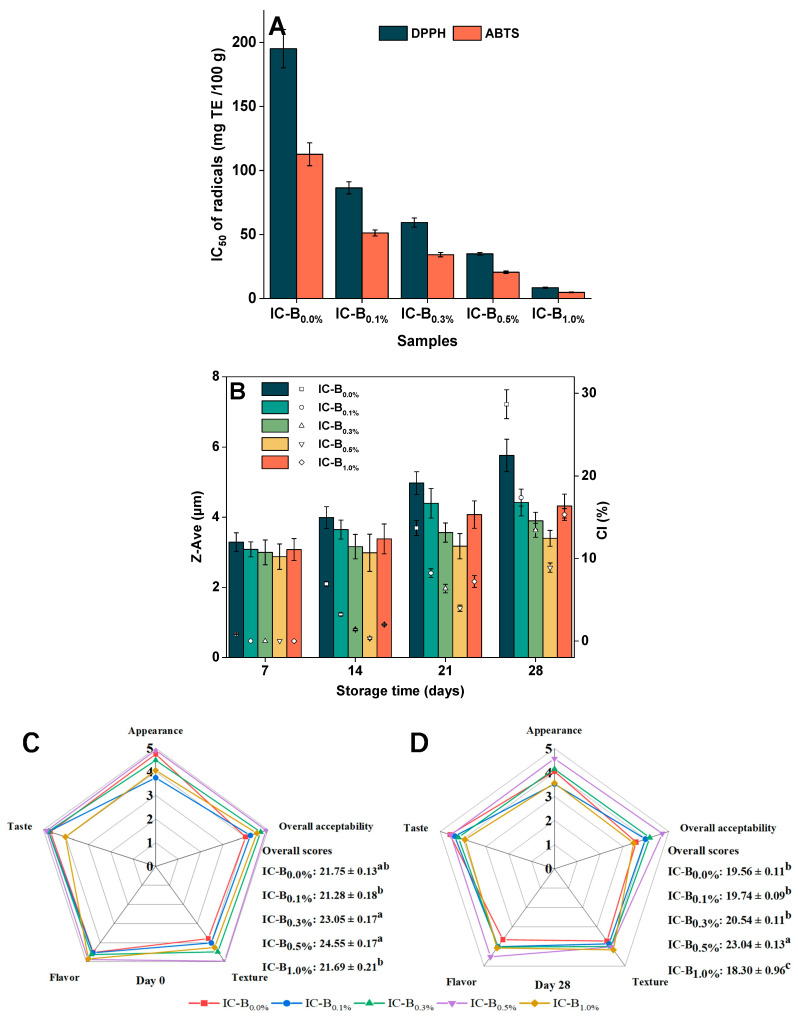
Antioxidant activities (**A**), storage physical stability (**B**), and sensory scores (**C**,**D**) of ice cream prepared with different levels of BMP_15Hz1.5h_. Means with different lowercase letters in the same row are significantly different (*p* < 0.05).

The addition of BMP_15Hz6h_ into the ice cream formula significantly improved DPPH and ABTS radical scavenging activities (Figure 3A) of IC-B samples (*p*  <  0.05). The IC_50_ values of DPPH and ABTS decreased from 195.13 mg TE/100 g to 8.47 mg TE/100 g and from 125.22 mg TE/100 g to 5.35 mg TE/100 g, respectively, as the added levels of BMP_15Hz6h_ increased from 0.1% to 1.0%. Blueberry peel has strong antioxidant activities due to its rich phenolics, anthocyanins, and other bioactive substances, and the total phenolics content is reported as about 55 mg of GAE/g dry weight [10]. Therefore, the strong free radical scavenging activities of IC-B samples are attributed to the presence of BMP_15Hz6h_, which has rich anthocyanins, as well as the antioxidants in camellia oil. Camellia oil has high antioxidant activity because it is rich in phenols, squalene, and vitamin E, but it is also easily oxidized because of its high content of polyunsaturated fatty acids [18]. The DPPH values of probiotic ice cream with blueberry puree [34] and camel milk ice cream with blueberry fruits [33] were reported in the range of 8.91–9.20% and 213–125 µg/mL (IC_50_), respectively.

### 3.5. Effects of BMP on Storage Stabilities of Ice Cream

#### 3.5.1. Storage Physical Stability

The physical storage stabilities were determined using Z-ave and CI (Figure 3B). As the added levels of BMP_15Hz6h_ increased, the Z-ave and CI values of IC-B samples decreased first during storage, reaching the lowest values at 0.5% BMP_15Hz6h_ addition, and then increased at 1.0% BMP_15Hz6h_ addition. The Z-ave and CI values of all IC-B samples increased with the extension of the storage time, but the increment in the values of Z-ave and CI was lower for IC-B samples with BMP_15Hz6h_ compared with ice cream without BMP_15Hz6h_. Also, after 7 days of storage, there was no significant difference in Z-Ave and CI values for all of the IC-B samples with BMP_15Hz6h_; nevertheless, with the extension of the storage time, IC-B_0.5%_ showed the lowest values of Z-Ave and CI values. The results suggested that a suitable level of BMP_15Hz6h_ added was required for the preparation of ice cream with high storage stability, which is in line with the results of Velasquezz-cock et al. [39], and that IC-B_0.5%_ showed the highest physical storage stability.

#### 3.5.2. Sensory Attributes

IC-B_0.0%_ and IC-B_0.5%_ had the highest appearance scores (*p* > 0.05), which were tempting creamy yellow and blueberry purple, respectively. No significant differences in the taste scores were observed among the samples, except for IC-B_1.0%_. Compared with IC-B_0.0%_, the texture scores of IC-B_0.1%_, IC-B_0.3%_, IC-B_0.5%_, and IC-B_1.0%_ increased by 5.80%, 18.21%, 31.13%, and 12.14%, respectively. The variation trend of the flavor and overall acceptability scores was similar to that of the texture score. Similar to our results, ice cream with an appropriate amount of blueberry puree [33,34] or fruit peel [7,8] incorporated could be desirable to consumers.

**Figure 4:**
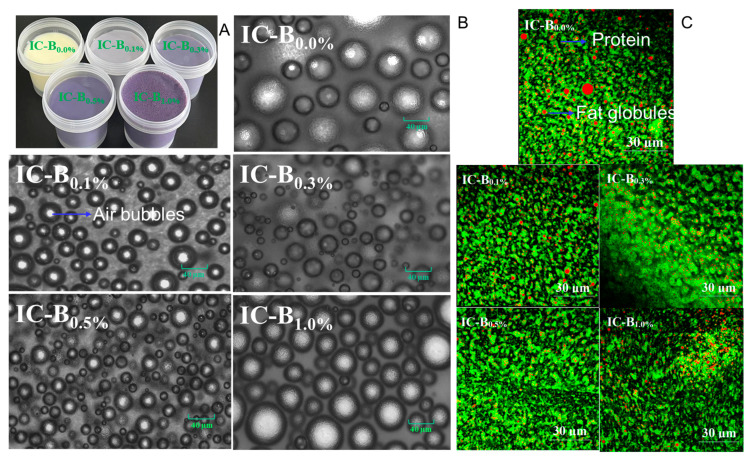
Visuals (**A**), optical microscopy images (**B**), and confocal micrographs (**C**) of ice cream prepared with different levels of BMP_15Hz1.5h_. (Red and green in (**C**) represent fat globules and protein granules, respectively.)

During storage, as the added levels of BMP_15Hz6h_ increased, the overall score value of IC-B samples increased first, reaching the highest value of 24.55 (at 0.5% BMP_15Hz6h_ addition), and then decreased to 21.69 (at 1.0% BMP_15Hz6h_ addition), which was similar to that of IC-B_0.0%_ (Figure 3B). On the last day of storage, the overall score of all samples decreased. IC-B_0.5%_ had the highest overall score, followed by IC-B_0.3%_, IC-B_0.1%_, IC-B_0.0%_, and IC-B_1.0%_ (Figure 3D), which agrees with the results of Z-ave and CI (Figure 3B) and suggests that the addition of 0.5% BMP_15Hz6h_ in ice cream mix may be a threshold level that is appealing for consumers. This may be because 0.5% BMP_15Hz6h_ not only gives the ice cream an intense color and various functional compositions (Table 3 and Figure 4) but also improves the texture due to its Pickering particles attribute, which helps air bubbles, protein, and fat globules distribute evenly (Figure 4).

### 3.6. Effects of BMP on Macro- and Micro-Structure of Ice Cream

The color of IC-B samples changed from milky yellow to deep purple red as the level of BMP_15Hz6h_ added increased from 0 to 1.0% (Figure 4A), wherein purple (at 0.5% BMP_15Hz6h_ addition) is the enjoyable color. The air bubbles in the IC-B_0.0%_ sample were big and sparse, which probably occurred due to the high fat content (10%) of camellia oil; nevertheless, more and smaller air bubbles were observed as the addition of BMP_15Hz6h_ increased to 0.5%, but the air bubbles became too big as the added level of BMP_15Hz6h_ reached 1.0% (Figure 4B). Many big red fat globules in the IC-B_0.0%_ sample could be seen in the visual field, but the fat globules gradually diminished and evenly distributed as the added level of BMP_15Hz6h_ was increased up to 0.5%; however, the size of the fat globules became large again (Figure 4C). Similar results were found by Bilbao-Sainz, Sinrod, Chiou, and McHugh [40], who added strawberry powder to the ice cream mix and found that an appropriate addition level was necessary to ensure the uniform distribution of air bubbles and fat globules in ice cream and the delectability of products. BMP_15Hz6h_ ameliorated the distribution of air bubbles and fat globules in ice cream in the following ways: (1) BMP_15Hz6h_ might serve as Pickering emulsifiers to enhance the interfacial properties of ice cream, (2) abundant dietary fiber with colloidal properties could stabilize oil in water, (3) abundant phenolics and anthocyanins act with protein further increase the viscosity, and (4) the pectin BMP and xanthan gum have the power to reinforce the gel structure, preventing bubble collapse in the process.

## 4. Conclusions

The results indicated that blueberry peel particles with Pickering attributes can be obtained via low-temperature ball milling, especially those ball-milled at 15 Hz for 6 h, and are good stabilizers for ice cream. The BMP_15Hz6h_ imparted a purple color and improved the antioxidant capacity and anthocyanin profile of the products. The use of substitute by-products and ingredients made it possible to obtain functional ice cream that can meet the demand of consumers who are looking for healthier ice cream with substitute ingredients.

## Figures and Tables

**Table 1:** Formulations of ice cream.

Ingredients (g/100 g)	IC-B_0.0%_	IC-B_0.1%_	IC-B_0.3%_	IC-B_0.5%_	IC-B_1.0%_
Skim milk	64.75	64.65	64.45	64.25	63.75
Sucrose	14	14	14	14	14
Skim milk powder	11	11	11	11	11
Camellia oil	10	10	10	10	10
Xanthan gum	0.25	0.25	0.25	0.25	0.25
BMP_15Hz1.5h_	0	0.1	0.3	0.5	1.0

IC-B, the ice cream prepared with different levels of BMP_15Hz1.5h_ added. The subscripts behind express the added level of BMP_15Hz1.5h_ in the ice cream mix.

**Table 2:** Effects of ball-milling frequency and ball milling time on size properties of blueberry peel particles.

Samples	D_10_ (μm)	D_50_ (μm)	D_90_ (μm)	Span	Φ (%)	SSA (m^2^/g)
CMP	29.83 ± 3.37 ^a^	210.28 ± 2.55 ^a^	547.68 ± 31.34 ^a^	2.46 ± 0.02 ^c^	13.60 ± 0.00 ^d^	0.11 ± 0.00 ^e^
BMP_15Hz1.5h_	5.43 ± 0.06 ^b^	23.12 ± 0.31 ^b^	60.95 ± 1.44 ^c^	2.40 ± 0.01 ^bc^	81.73 ± 0.03 ^c^	0.52 ± 0.01 ^d^
BMP_15Hz3.0h_	4.01 ± 0.01 ^c^	17.36 ± 0.05 ^c^	50.95 ± 0.26 ^d^	2.70 ± 0.02 ^c^	92.38 ± 0.02 ^ab^	0.67 ± 0.00 ^b^
BMP_15Hz6.0h_	2.62 ± 0.16 ^e^	15.00 ± 0.14 ^e^	36.00 ± 0.88 ^f^	2.22 ± 0.03	96.38 ± 0.01 ^a^	1.01 ± 0.05 ^a^
BMP_25Hz1.5h_	3.85 ± 0.25 ^c^	17.64 ± 3.44 ^c^	75.36 ± 6.12 ^b^	4.05 ± 0.01 ^a^	91.88 ± 0.02 ^b^	0.63 ± 0.03 ^bc^
BMP_25Hz3.0h_	3.70 ± 0.12 ^cd^	16.18 ± 1.22 ^cd^	48.75 ± 8.31 ^d^	2.78 ± 0.02 ^bc^	94.43 ± 0.01 ^a^	0.68 ± 0.04 ^b^
BMP_25Hz6.0h_	3.54 ± 0.01 ^d^	15.12 ± 0.11 ^de^	39.92 ± 0.31 ^e^	2.41 ± 0.02 ^c^	96.12 ± 0.01 ^a^	0.71 ± 0.00 ^b^

CMP, commonly milled particles of blueberry peel; BMP, ball-milled particles of blueberry peel. The subscripts behind express the ball milling frequency and ball milling time, respectively; Φ, cell wall breakage ratio. Means in the same column with different superscript letters are significantly different at *p* < 0.05.

**Table 3:** Compositions and percentage of anthocyanins in blueberry peel particles.

No.	Anthocyanins	Retention Time (min)		[M-H]^−^(*m*/*z*)	Fragment Masses	Percentage (%)	
		CMP	BMP_15Hz1.5h_			CMP	BMP_15Hz1.5h_
1	Cy	16.83	16.84	287.05	449.11, 287.06	0.002 ^b^	0.006 ^a^
2	Cy-3,5-O-dig	1.99	1.99	611.16	463.12, 625.18, 301.07	0.005	0.006
3	Cy-3-O-gal/glu	5.57	5.61	449.11	287.06	8.101	8.102
4	Cy-3-O-ara	6.57	6.59	419.10	287.06	2.245	2.245
5	Cy-3-O-sop	11.75	11.74	611.16	317.07	0.099	0.096
6	Cy-3-O-rut	13.05	13.07	595.17	287.06	0.015	0.014
Total percentage						10.467	10.469
7	Dp	13.22	13.22	303.05	317.07	0.752	0.764
8	Dp-3-O-(6″-p-coumaryl)-glu	16.42	16.42	611.14	303.05	0.070	0.065
9	Dp-3-O-(6-O-acetyl)-glu	14.51	14.53	507.11	303.05	0.373	0.366
10	Dp-3-O-ara	13.22	13.21	435.09	303.05	0.397	0.396
11	Dp-3-O-gal/glu	12.23	12.21	465.10	303.05	1.869	1.872
12	Dp-3-O-rha	5.57	5.58	449.11	317.07	8.064	8.061
13	Dp-3-O-rut	1.99	1.99	611.16	317.07	0.005	0.005
Total percentage						11.530	11.529
14	Mv-3-O-(6″-acetyl)-gal/glu	10.65	9.92	535.14	331.08, 493.13	1.883	1.885
15	Mv-3-O-(6″-malonyl)-glu	9.51	9.51	579.13	331.08, 493.13	0.138	0.135
16	Mv-3-O-(6-O-p-coumaryl)-O-glu	12.37	12.38	639.17	331.08	0.094	0.092
17	Mv-3-O-ara	8.15	8.16	463.12	331.08	15.248	15.248
18	Mv-3-O-gal/glu	7.42	7.43	493.13	331.08	24.630	24.633
19	Mv-3,5-O-dig	13.09	13.10	655.19	287.06	0.004	0.004
Total percentage						41.997	41.997
20	Pg	9.07	9.06	271.06	301.07	0.001 ^b^	0.003 ^a^
21	Pg-3,5-O-dig	13.05	13.05	595.17	303.05	0.015	0.013
22	Pg-3-O-gal/glu	7.84	7.85	433.11	493.13, 331.08	1.297	1.296
Total percentage						1.313	1.312
23	Pn	15.76	15.76	301.07	303.05	0.002 ^b^	0.005 ^a^
24	Pn-3-O-(6″-acetyl)-gal/glu	10.49	10.44	505.13	331.08	0.265	0.262
25	Pn-3-O-ara	7.84	7.85	433.11	301.07, 463.12	1.297	1.297
26	Pn-3-O-gal/glu	8.15	8.16	463.12	433.11, 271.06	15.248	15.249
27	Pn-3-O-sop-5-O-glu	8.89	8.88	787.23	271.06	0.002	0.001
Total percentage						16.814	16.814
28	Pt	16.84	16.85	317.06	303.05	0.018 ^b^	0.025 ^a^
29	Pt-3-O-rut-5-O-glu	8.89	8.88	787.23	317.07, 479.12, 625.18	0.002	0.001
30	Pt-3-O-(6″-acetyl) gal/glu	9.26	9.27	521.13	301.07	0.887	0.883
31	Pt-3-O-(6″-malonyl)-glu	14.86	14.88	565.12	317.07, 479.12	0.063	0.060
32	Pt-3-O-gal/glu	6.30	6.29	479.12	317.07, 479.12	8.581	8.585
33	Pt-3-O-rut	13.00	12.99	625.18	317.07, 479.12	0.212	0.212
34	Pt-3-O-p-coumaroyl-O-glu	16.65	16.66	625.15	317.07, 479.12	0.010 ^a^	0.005 ^b^
35	Pt-3-O-ara	5.57	5.59	449.11	301.07	8.101	8.101
Total percentage						17.874	17.872

*m*/*z*, mass-to-charge ratio; Cy, cyanidin; dig, diglucoside; gal, galactoside; glu, glucoside; ara, arabinoside; sop, sophoroside; rut, rutinoside; Pt, petunidin; Dp, delphinidin; rha, rhamnoside; Mv, malvidin; Pn, peonidin; Pg, pelargonidin. For other abbreviations, see Table 2. Means in the same line with different superscripts are significantly different at *p* < 0.05.

**Table 4:** Physical properties of ice cream prepared with different levels of BMP_15Hz1.5h_.

Samples	Overrun (%)	Firmness (N)	First Dripping Time (min)	Melting Rate (%/min)
IC-B_0.0%_	32.99 ± 0.85 ^c^	33.15 ± 2.16 ^a^	14.16 ± 0.93 ^c^	3.13 ± 0.82 ^a^
IC-B_0.1%_	36.51 ± 2.01 ^b^	25.97 ± 1.79 ^b^	17.43 ± 0.89 ^b^	2.84 ± 0.03 ^b^
IC-B_0.3%_	38.75 ± 2.34 ^ab^	19.31 ± 1.44 ^c^	18.54 ± 1.01 ^ab^	2.83 ± 0.08 ^b^
IC-B_0.5%_	45.13 ± 3.06 ^a^	19.03 ± 1.18 ^c^	20.18 ± 1.07 ^a^	2.71 ± 0.02 ^b^
IC-B_1.0%_	38.22 ± 2.92 ^ab^	24.41 ± 1.42 ^b^	18.00 ± 1.10 ^ab^	2.77 ± 0.11 ^b^

Abbreviations: see Table 1. Means in the same row with different superscripts are significantly different at *p* < 0.05.

## Data Availability

The original contributions presented in the study are included in the article, further inquiries can be directed to the corresponding author.
